# Effects of acute severe hypobaric hypoxia on gut-muscle axis in rats

**DOI:** 10.1007/s13105-026-01213-y

**Published:** 2026-07-25

**Authors:** Garoa Santocildes, Karenia Lorenzo, José Magalhães, Susana Amézqueta, Teresa Pagès, Josep Lluís Torres, Ginés Viscor, Sara Ramos-Romero, Joan Ramon Torrella

**Affiliations:** 1 Department of Cell Biology, Physiology and Immunology, Faculty of Biology, University of Barcelona, Avinguda Diagonal, 643, 08028 Barcelona, Spain; 2 Department of Sport Biology, Faculty of Sport, Research Centre in Physical Activity, Health and Leisure (CIAFEL), University of Porto, Porto, Portugal; 3 Departament d’Enginyeria Química i Química Analítica and Institut de Biomedicina (IBUB), Universitat de Barcelona, Barcelona, Spain; 4 Department of Biological Chemistry, Institute of Advanced Chemistry of Catalonia (IQAC-CSIC), Barcelona, Spain; 5 Maria de Maeztu Unit of Excellence, Nutrition & Food Safety Research Institute (INSA-UB), Santa Coloma de Gramenet, Spain

**Keywords:** Hypobaric hypoxia, Gut–muscle axis, Intestinal morphology, Microbiota, Mitochondrial respiration, Altitude physiology.

## Abstract

Acute exposure to high-altitude hypobaric hypoxia (HH) disrupts physiological homeostasis. While gastrointestinal and neurological disturbances are its main manifestations, muscle function is also affected. However, its short-term effects on the gut–muscle axis remain poorly understood. The present study investigated the impact of a 4-hour exposure to simulated altitude (5,000 m) on intestinal morphology, gut microbiota composition, short-chain fatty acid (SCFA) levels, and skeletal muscle mitochondrial function in male Sprague-Dawley rats. Despite the severity of the hypoxic stimulus, biometric and hematological parameters remained largely unchanged, suggesting preserved systemic stability. Histological analysis revealed a significant reduction in intestinal crypt depth, indicating early structural alterations, while villus morphology and goblet cell counts remained unchanged. Microbiota profiling showed a decrease in Enterobacteriales abundance, while other bacterial groups and SCFA concentrations remained stable, pointing to limited microbial shifts under acute conditions. In skeletal muscle, reactive oxygen species (ROS) production increased under complex I-supported respiration while mitochondrial respiration was maintained across all respiratory states. This suggests a qualitative shift in redox balance. Collectively, these findings indicate that acute HH triggers early localized responses in intestinal and muscular tissues without inducing widespread systemic disruption, highlighting the resilience of the gut–muscle axis to short-term hypoxic stress. Longer or repeated exposures may be required to elicit broader physiological adaptations. This study contributes to understanding the early effects of altitude-related hypoxia and underscore the importance of exposure duration in shaping host responses.

## Key Points

Acute hypobaric hypoxia did not alter body weight nor hematological parameters.

The intestine exhibit structural changes under acute hypoxia.

Acute hypobaric hypoxia produce reduction of caecal Enterobacteriales abundance.

Caecal SCFA levels remained stable after acute hypobaric hypoxia.

Acute hypoxia produce increases in ROS production in skeletal muscle.

There were no changes in mitochondrial respiration after acute hypobaric hypoxia.

## Introduction

 Normoxia refers to normal oxygen levels in organs, tissues, and cells which ensures an adequate oxygen supply to sustain physiological functions. In contrast, hypoxia occurs when the oxygen supply is insufficient to meet the cellular demand [1, 2]. However, hypoxic conditions are not necessarily pathological. Some factors such as diet, exercise, or altitude can lead to physiological hypoxia in cells [2].

Acute exposure to high altitude is defined as the sudden ascent from lowland areas to plateau areas situated higher than 2,500 m in altitude [3–6]. High-altitude environments are characterised by low barometric pressure, resulting in a low partial pressure of oxygen (hypobaric hypoxia, HH) and, ultimately, a reduced oxygen availability at the tissue level. This environmental stressor can disrupt homeostasis and produce some health impairments under the high-altitude illnesses umbrella, from acute mountain sickness, generally mild and transient, to the more severe and even life-threatening high-altitude cerebral oedema and high-altitude pulmonary oedema [4, 5, 7]. Although some of these effects are often transient and may promote physiological resilience to subsequent stress, they can compromise performance and contribute to chronic health disease or lead to chronic sequelae in some individuals [8–12]. Though the most recognizable symptoms of acute mountain sickness are neurological and digestive (headache, anorexia, malaise, vomiting, etc.), there is also musculoskeletal involvement: dyspnea, fatigue, and, in severe forms, even alterations in motor control such as ataxia [4, 5, 7].

Within the human body, oxygen supply is regulated based on the metabolic demands of individual tissues; thus, hypoxic stress varies across tissues, and not all respond similarly to decreases in atmospheric oxygen pressure or oxygen availability [13]. However, all nucleated mammalian cells are sensitive to changes in oxygen tension and respond to maintain homeostasis and ensure survival [14, 15]. The heterodimeric protein HIF-1 is the principal cellular mediator of the hypoxic response. The HIF-1α subunit is highly sensitive to oxygen levels and, under normoxic conditions, undergoes hydroxylation followed by proteasomal degradation. However, under hypoxic conditions, HIF-1α degradation is inhibited, leading to its accumulation. Subsequently, HIF-1α translocates to the nucleus, where it activates the transcription of more than 100 genes involved in the cellular response to hypoxic stress [14, 15].

Certainly, some organs and tissues are physiologically under conditions of relative hypoxia [2]. In the case of the intestine, it displays a highly vascularized and well-oxygenated subepithelial mucosa, while on the opposite side it presents a poorly oxygenated lumen. Moreover, the gastrointestinal (GI) tract of rats naturally exhibits steep oxygen gradients, both horizontally (from the intestinal wall layer to the lumen) and vertically (from the duodenum to the colon) [2, 13, 16, 17]. Horizontally, oxygen gradients range from around 4–8% in the subepithelial region of the small intestine to 2–4% at the villus tips and fall to below 2% within the intestinal lumen. Additionally, this tissue experiences daily fluctuations in oxygen perfusion related to feeding cycles, with higher oxygen levels observed postprandially compared to fasting conditions [13]. Therefore, a hypoxic environment at the intestinal level is not inherently detrimental and is, in fact, crucial to ensure essential physiological processes [2].

However, this physiological hypoxia in the GI tract can be exacerbated by acute or chronic stressors that reduce oxygen availability, such as intense exercise or high-altitude exposure [16]. It has been previously reported that rats exposed to environmental acute hypoxia (15% O_2_) presented reduced oxygen partial pressure (PO_2_) in both the serosal and mucosal layers, suggesting that oxygen levels in the intestine are modified by environmental hypoxic exposure [17]. During acute hypoxia, an increase in sympathetic tone leads to vasoconstriction and intestinal ischemia [18], resulting in energy depletion and tissue acidosis, which can injure the intestinal barrier [18]. The damaged gastric intestinal barrier may increase gastric acid secretion and, consequently, contribute to acute gastrointestinal injury [19]. GI affections are commonly reported during high altitude exposure (> 2,500 m); in fact, GI symptoms are a part of the 2018 Lake Louise Acute Mountain Sickness Score [20], with anorexia, nausea, and vomits, being the most common symptoms [3, 4, 18]. Furthermore, peptic ulcers have been observed within the first 2–4 days after ascents to high altitudes (5,100 m) [18], due to the incapability of the individuals to acclimatize to that environment [3]. These symptoms tend to appear rapidly and show a dose-response relationship, that is, more severe hypoxia elicits stronger responses [3]. Although the disruption of the intestinal barrier has been proposed as a potential underlying mechanism to explain GI complications [18], intestinal defence is not limited to the mechanical barrier. Together with this mechanical component, the biological, chemical and immune barriers constitute a complete defence system. In this context, the gut microbiota, acting as a biological barrier, is considered a key “organ” contributing to the host’s health [3] and is directly influenced by hypobaric hypoxia [19]. Under systemic hypoxia conditions, colonocytes seem to adapt their metabolism by decreasing their oxygen consumption, thereby allowing oxygen to be redistributed to more vital organs and tissues. Paradoxically, this metabolic shift in colonocytes increases the concentration of respiratory electron acceptors (O_2_ and NO_3_^–^), which diffuse into the intestinal lumen. As a result, the microbial community changes from mostly obligate anaerobes towards an increase of facultative anaerobic bacteria [21]. Exposure to acute HH induces changes in the composition of the gut microbiota, depending on altitude and duration of exposure. Some of the bacterial orders most likely to be affected by HH are Lactobacillales (phylum Firmicutes), Enterobacteriales (class γ-Proteobacteria), and Bifidobacteriales (phylum Actinobacteria) [12, 22]. Changes in the relative abundance of *Prevotella* have also been documented [3]. These microbial alterations trigger an immediate response from the host’s immune system.

Muscle health is pivotal in whole-body metabolism, as skeletal muscle accounts for up to 40% of total body mass and is the major site of insulin-stimulated glucose uptake [23]. Skeletal muscle is also one of the tissues affected by hypoxia. Nevertheless, muscle can maintain its homeostasis at a low oxygen percentage (between 2 and 10%), as it is naturally subjected to oxygen restriction during various physiological situations, such as exercise [1, 15, 24]. During hypoxia, mitochondria, being the major consumers of oxygen, are significantly impacted by the drop in oxygen availability [25]. Specifically, hypoxia modifies key mitochondrial processes including fusion and fission dynamics, mitophagy and oxidative phosphorylation (OXPHOS) [25]. It is well established that hypoxia reduces OXPHOS, largely due to a decrease in the production of reduced equivalents (NADH, FADH_2_) from the tricarboxylic acid (TCA) cycle under low oxygen conditions. However, OXPHOS can adapt to hypoxia through continuous remodelling of the electron transport chain (ETC) and adjustments in the enzymatic activity of the TCA cycle. On the other hand, paradoxically, during acute hypobaric hypoxia exposure, there is an additional production of free radicals, resulting from different mechanisms activated in response to oxygen deprivation [26].

In recent decades, the existence of a gut-muscle axis has been demonstrated, highlighting the impact of gut microbiota on muscle mass, muscle quality and muscle function. Gut microbiota and host mitochondria share a close and dynamic relationship, with bidirectional communication occurring between them. This interaction primarily involves signalling from the gut microbiota to the host mitochondria, and then back from host mitochondria to the gut mediated through endocrine, immune and humoral pathways [27]. The metabolites produced by gut microbiota, such as short-chain fatty acids (SCFA) and secondary bile acids, can influence host mitochondrial function at different organs by modulating energy production, mitochondrial biogenesis, redox balance and inflammatory signalling pathways [27]. It has been shown that lacking gut microbiota directly reduces mitochondrial DNA content and leads to impaired mitochondrial biogenesis and oxidative capacity in skeletal muscle [23]. Therefore, it has been well-established that gut microbiota plays a critical role in the maintenance of skeletal muscle health. Conversely, mitochondrial function may affect gut microbiota composition and its activity by initiating innate immune responses and influencing intestinal cells, including immune cells, epithelial cells, and enterochromaffin cells [27].

Each year, millions of people are acutely exposed to hypobaric hypoxia environments due to occupational demands (such as military, border control, astronomy, mining operations, mountain rescue teams) or recreational activities (such as sightseeing, skiing, climbing, trekking) [4, 6, 28]. The risk of suffering from acute mountain sickness is variable and depends on the altitude and speed of ascent, but it is much more frequent above 2,500 m [29]. Thus, our study in a rodent model, aims to evaluate the effects of acute exposure to severe hypobaric hypoxia on intestinal, gut microbiota, and muscular parameters, with particular emphasis on early responses within the gut–muscle axis.

## Materials and methods

### Animals

A total of 19 male Sprague-Dawley rats from Envigo (Envigo RMS Spain S.L.; Sant Feliu de Codines, Barcelona, Spain), aged 11 weeks, were used in the study. Animals were housed (*n* = 2 per cage) under controlled conditions of humidity (60%) and temperature (22 ± 2 °C) with a 12 h light-12 h dark cycle and free access to water and food. All procedures were strictly adhered to the European Union guidelines for the care and management of laboratory animals and were authorised by the regional Catalan authorities (ref. no 1332/2022), as approved by the Spanish CSIC Subcommittee of Bioethical Issues.

### Experimental groups and acute hypobaric hypoxia exposure

Rats were randomly divided into 2 groups (*n* = 9–10 per group): (1) Control group (CTR), maintained in normoxia, and (2) Acute hypoxia group (HYP), submitted to a single acute hypobaric hypoxia (AHH) exposure of 5,000 m of simulated altitude. Hypobaric hypoxia was created using a hypobaric chamber (450 L), made of polymethyl methacrylate plastic walls. Low pressure into the chamber was produced by a vacuum pump (TRIVAC D5E; Leybold, Köln, Germany) by regulating the airflow rate at the inlet with a micrometric valve. Inner pressure was controlled by two differential pressure sensors (ID 2000; Leybold, Köln, Germany) connected to a vacuum controller (Combivac IT23; Leybold, Köln, Germany). The target pressure of 405 torr (equivalent to 5,000 m of altitude) was achieved steadily over ∼15 min. Once this desired level was reached, the internal barometric pressure of the chamber was regulated and maintained by the control system for 4 h. At the end of the session, pressurization to normal barometric pressure was gradually restored over ∼15 min.

### Data and sample collection

Animals from both experimental groups were subjected to a standardized 15-hour overnight fast (with free access to water). Subsequently, the HYP group was exposed to acute hypobaric hypoxia (AHH), whereas the CTR group remained at rest under normoxic conditions. Immediately after both hypoxic and control protocols, animals were anaesthetised intraperitoneally with ketamine and xylazine (80 and 10 mg per kg body weight, respectively). Then, a blood sample (7–9 mL) was drawn from the abdominal cava vein and immediately the soleus (SOL) and gastrocnemius (GAS) muscles, the heart, the perigonadal adipose tissue (PAT), kidneys, spleen, caecum, small intestine (jejunum and ileum) and liver were excised, and animals were killed by exsanguination. All the organs and samples were carefully weighed and stored at -80 °C. One portion of the small intestine was fixed in 4% formalin for histological assays. A fresh aliquot of the blood sample was analysed by a haematological analyser (Coulter Spincell 3; Spinreact, Barcelona, Spain).

### Intestine histology

After being removed, the jejunum from the animals was washed with saline and fixed in buffered formalin 10% (pH 7) at room temperature for 24 h, rinsed with PBS, dehydrated with ethanol 95% and embedded in paraffin. Paraffin blocks were cut with a microtome into 5 μm-thick sections and then followed a dehydration protocol: 5 min in xylene (x2), 1 min in ethanol 100%, 5 min in ethanol 75%, 10 min in ethanol 50% and rinsed in distilled water for 5 min. Afterwards, the slides were stained with haematoxylin-eosin (2 min in haematoxylin and 1 min in 1% eosin), dehydrated (30 s in ethanol 90% and 30 s in ethanol 100%), cleared in xylene and mounted with DPX medium. Images of intestine sections were acquired with a light microscope (BX61; Olympus, Tokyo, Japan) connected to a digital camera (DP70, Olympus). The analysis was carried out using ImageJ software (v. 1.51n; National Institutes of Health, Bethesda, MD, USA). The average of 10 villi measurements of each animal was used to perform the analyses.

### Caecal bacterial subgroups

The levels of total bacteria and several bacterial subgroups (Table 1) were estimated from caecal DNA by quantitative real-time PCR (qPCR). First, total DNA of the samples was extracted using a commercial kit (HigherPurity™ Stool DNA Isolation Kit; Canvax, Valladolid, Spain), and its concentration was quantified using a NanoDrop 2000 Spectrophotometer (Thermo Fisher Scientific, Cornella de Llobregat, Spain). Subsequently, all DNA samples were diluted up to 20 ng/µL. Samples were run in triplicate, and each qPCR well contained a total volume of 5 µL of reaction mixture: 2.5 µL of SYBR green Supermix, 0.25 µL of forward and reverse primers, 1 µL of DNA sample, and 1 µL of DEPC water. In all qPCR plates, a non-template control (H_2_O) and a positive control (Table 1) were run in parallel to the samples. CFX384™ Real-Time System (Bio-Rad Laboratories; Hercules, CA, USA) was employed to carry out the qPCR in Hard-ShellR 384-Well PCR Plates (HSP3801, Bio-Rad Laboratories).

**Table 1:** Quantitative real-time PCR primers and conditions

Target bacterial subgroup	Sequence (5’-3’)	Annealing Temp. (ºC)	Positive Control
Total Bacteria	F: ACT CCT ACG GGA GGC AGC AGT R: ATT ACC GCG GCT GCT GGC	65	(*)
Firmicutes	F: CTG ATG GAG CAA CGC CGC GT R: ACA CYT AGY ACT CAT CGT TT	52	*Ruminococcus productus*
γ-Proteobacteria	F: GCT CGT GTT GTG AAA TGT TGG R: CGT AAG GGC CAT GAT GAC TTG	54	*Escherichia coli*
Actinobacteria	F: TAC GGC CGC AAG GCT A R: TCR TCC CCA CCT TCC TCC G	54	*Bifidobacterium* *longum*
Lactobacilliales	F: AGC AGT AGG GAA TCT TCC A R: CAC CGC TAC ACA TGG AG	60	*Lactobacillus acidophylus*
Enterobacteriales	F: ATG GCT GTC GTC AGC TCG T R: CCT ACT TCT TTT GCA ACC CAC T	60	*Escherichia coli*
Bifidobacteriales	F: CTC CTG GAA ACG GGT GG R: GGT GTT CTT CCC GAT ATC TAC A	55	*Bifidobacterium* *longum*
*Prevotella*	F: CAG CAG CCG CGG TAA TA R: GGC ATC CAT CGT TTA CCG T	60	*Prevotella copri*

* Positive control of total bacteria was the same as those the result was rated with

The qPCR cycling conditions were: 10 s at 95 °C, then 45 cycles of 5 s at 95 °C, 30 s at primer-specific annealing temperature (Table 1), and 30 s at 72 °C (extension). After amplification, to assess the specificity of the qPCR reactions, the melting curve analysis was carried out: 2 s at 95 °C and 15 s at 65 °C, followed by continuous increase of the temperature up to 95 °C at a rate of 0.11 °C/s, with five fluorescence readings per °C. The relative DNA abundance of each bacterial subgroup was calculated from the second derivative maximum of their respective amplification curves (Cp, calculated in triplicate), by considering Cp values to be proportional to the dual logarithm of the inverse of the specific DNA concentration, according to the equation: [DNA_a_] / [DNA_b_] = 2^Cpb−Cpa^ [30]. Total bacteria presence was normalised as 16 S rDNA gene copies per mg of wet caecal content (copies per mg).

### Caecal short-chain fatty acids

Short Chain Fatty Acids (SCFAs) were analysed in caecal content by gas chromatography using the previous method proposed by Schwiertz [31], incorporating some modifications. For that, a solution (1.5 mL) containing the internal standard 2-ethylbutiric acid (6.67 mg/L) and oxalic acid (2.97 g/L) in acetonitrile/water (3:7) was added to freeze-dried samples (∼50 mg dry matter) and the SCFAs were extracted using a rotating mixer (10 min). Then the suspension was centrifuged (5 min at 12,880 g) and the supernatant filtered through a 0.45 μm nylon filter. From this, aliquots of supernatant (0.7 mL) were diluted in acetonitrile/water (3:7) up to 1 mL. Finally, SCFAs of the samples were analysed using a Trace 2000 gas chromatograph coupled to a flame ionisation detector (ThermoFinnigan; USA) equipped with an Innowax 30 m 530 μm x 1 μm capillary column (Agilent; USA). Helium, with a linear velocity of 5 mL/mi, was used as the carrier gas. GC oven temperature was programmed as follows: 80 °C (hold 1 min) to 120 °C, at 15 °C/minute (hold 4 min) to 130 °C, at 5 °C/min (hold 4 min) to 235 °C, at 8 °C/min (hold 4 min). Flame ionisation detection was performed at a base temperature of 240 °C. Calibration curves were prepared using seven matrix-matched standards covering the working concentration range. Chrom-Card software was used for data analysis.

### High-resolution respirometry: skeletal muscle mitochondrial respiration and reactive oxygen species production

After being excised, the right SOL muscle of the rats was immediately placed in cold Biopsy Preservation Solution (BIOPS). Next, muscle fibres were mechanically separated under a microscope, and individual fibres were permeabilised with saponin (20-min incubation, 50 µg/mL saponin in BIOPS). Between 10 and 20 mg of wet fibre mass was used for each animal assay.

After tissue preparation, skeletal muscle mitochondrial respiration and hydrogen peroxide (H_2_O_2_) production were simultaneously measured in the permeabilized fibers by high-resolution respirometry using an Oxygraph-2k (O2k, Oroboros Instruments, Austria) combined with the Fluorescence-Sensor Green of the O2k-Fluo LED2-Module for H₂O₂ detection with Amplex UltraRed^®^. A previously established substrate-uncoupler-inhibitor titration protocol (SUIT-009, htt://wiki.oroboros.at/index.php/SUIT-009_AmR_ce-pce_D019) was applied: LEAK respiration supported by NADH-linked substrates through Complex I (N, pyruvate – 5 mM)) was measured in the absence of ADP, followed by OXPHOS capacity through Complex I [OXPHOS (N)] after ADP addition (2.5 mM). Succinate (10 mM) was then added to stimulate convergent electron flow through Complexes I and II [OXPHOS (NS)]. Complex I was inhibited with rotenone (0.5 µM) to assess OXPHOS through Complex II alone [OXPHOS (S)]. Residual oxygen consumption (ROX) was determined after Complex III inhibition with antimycin A (2.5 µM) and subtracted from all respiratory states. All substrates and inhibitors were added at a saturated concentration.

Mitochondrial respiration was expressed as mass-specific respiration, that is, normalised by muscle wet weight per second (pmol O_2_ · s^− 1^ · mg^− 1^). The O2K-software Datlab 7.4 was used for real-time data acquisition and for further data analysis.

### Statistical analysis

GraphPad Prism 5 (GraphPad Software, Inc., San Diego, CA, USA) software was used to carry out all data statistical analysis and to plot the figures. Results are expressed as mean values with their standard deviation (SD). The normal distribution and homoscedasticity of variance were evaluated using the Shapiro-Wilk and Levene´s test, respectively. The statistical significance when comparing CTR and HYP groups was determined by means of the Student´s *t*-test. Differences were considered significant when *p* < 0.05. Figures are presented as box-and-whisker plots. The box represents the interquartile range and shows the first and the third quartiles separated by the median; the mean value is indicated inside the box by a cross or a dot. Whisker endpoints represent the minimum and maximum values.

## Results

### Biometric and haematological responses to acute hypobaric hypoxia

Table 2 shows the body weight and organ proportions of rats after exposure to acute hypobaric hypoxia. Data from body weight, and the weights of the SOL and GAS muscles, PAT, caecum, kidneys, spleen, liver and heart were collected. No statistically significant differences were observed between CTR and HYP animals across any of the examined tissues.

**Table 2:** Effects of acute exposure to hypobaric hypoxia on rats´ body and organ weight biometrical parameters

	CTR		HYP		
	Mean	SD	Mean	SD	*P* value
Body Weight (g)	499	11	506	10	*p* = 0.780
Soleus (% BW)	0.094	0.002	0.096	0.002	*p* = 0.774
Gastrocnemius (% BW)	1.28	0.02	1.25	0.03	*p* = 0.368
Caecum (% BW)	0.496	0.046	0.467	0.031	*p* = 1.000
Kidney (% BW)	0.580	0.016	0.585	0.014	*p* = 0.967
Spleen (% BW)	0.258	0.010	0.247	0.010	*p* = 0.368
PAT (% BW)	1.20	0.06	1.50	0.13	*p* = 0.065
Liver (% BW)	2.72	0.16	2.66	0.07	*p* = 1.000
Heart (% BW)	0.331	0.008	0.310	0.009	*p* = 0.112

Data are presented as mean ± standard deviation. *BW* body weight, *CTR* control group, *HYP* acute hypobaric hypoxia group, *PAT *perigonadal adipose tissue. *n* = 9–10 per group. Statistical analysis was performed using Student’s t-test to compare CTR and HYP groups. Differences were considered statistically significant when *p* < 0.05

**Fig. 1:**
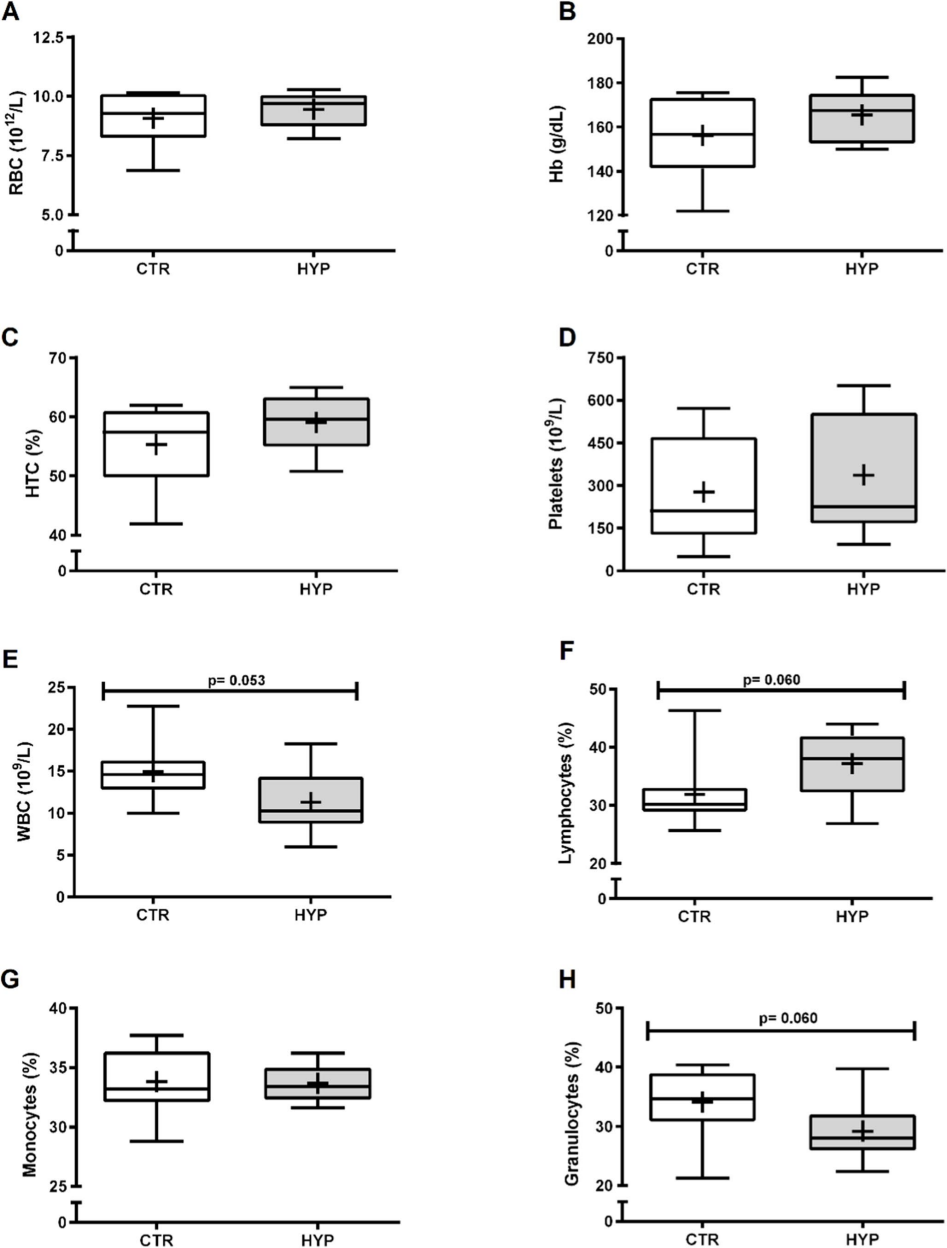
Effects of acute exposure to hypobaric hypoxia on haematological parameters. (**A**) Red Blood Cells (RBC); (**B**) Haemoglobin (Hb); (**C**) Haematocrit (HTC); (**D**) Platelets; (**E**) White Blood Cells (WBC); (**F**) Lymphocytes; (**G**) Monocytes; (**H**) Granulocytes. CTR: Control group; HYP: acute hypobaric hypoxia group. *n* = 9–10 per group. Statistical analysis was performed using Student’s t-test to compare CTR and HYP groups. Differences were considered statistically significant when *p* < 0.05. The box represents the interquartile range, showing the first and third quartiles separated by the median; the mean value is indicated inside the box by a cross. Whisker endpoints represent the minimum and maximum values

In Fig. 1, haematological parameters of the animals are presented. No statistical differences were observed in red blood cells (RBC), haemoglobin (Hb) and haematocrit (HTC) (*p* = 0.604; *p* = 0.269; *p* = 0.242, respectively) (Fig. 1A-C) after 4 h of hypobaric hypoxia exposure. In the same line, no changes between groups were found in the hematimetric parameters MCV (CTR = 62.8 ± 3.0 vs. HYP = 62.6 ± 2.2, *p* = 0.757), MCH (CTR = 17.6 ± 0.5 vs. HYP = 17.5 ± 0.6, *p* = 0.790) and MCHC (282 ± 10 vs. 280 ± 8, *p* = 0.653) (not shown). Moreover, no differences were observed in platelet count (*p* = 0.487) (Fig. 1D).

Regarding leukocytes, a not significant reduction were observed in total white blood cells (WBC) after hypoxic exposure (14.9 ± 3.5 vs. 11.3 ± 3.8, *p* = 0.053) (Fig. 1E). Considering the different types of WBC, a non-significant increase was observed in lymphocytes (32.0 ± 5.6 vs. 37.2 ± 5.6, *p* = 0.060) but no changes were appreciated in monocytes (33.8 ± 2.8 vs. 33.6 ± 1.5, *p* = 0.806). Finally, a non-significant reduction was seen in granulocytes (34.1 ± 5.9 vs. 29.2 ± 5.0, *p* = 0.060) (Fig. 1E-H).

### Intestinal and gut microbiota responses to acute hypobaric hypoxia exposure

#### Intestinal morphology analysis

**Fig. 2:**
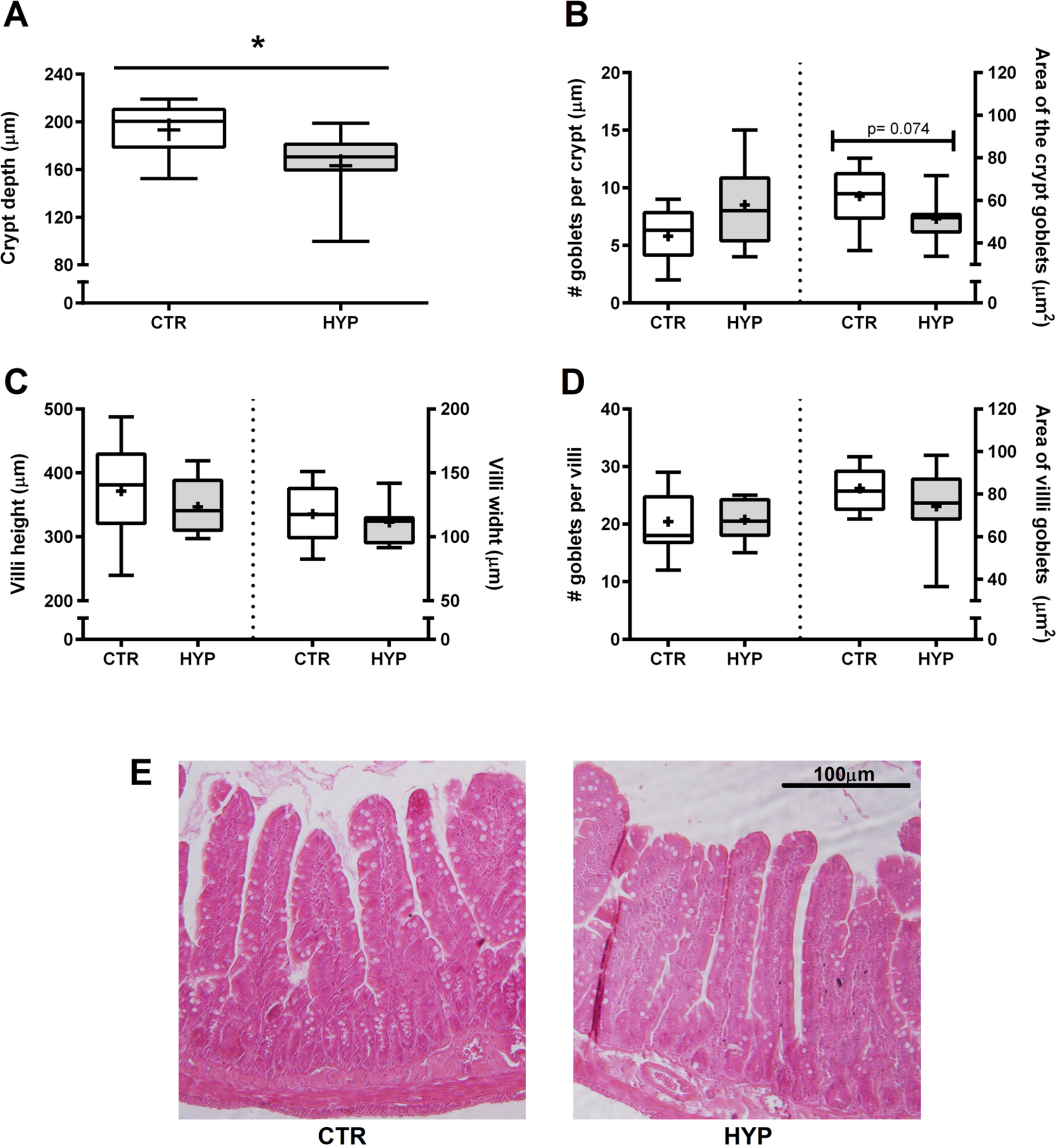
Effects of acute exposure to hypobaric hypoxia on intestinal histology. (A) Crypt depth; (B) Number (#) of goblets per crypt and area of the crypt goblets; (C) Villi height and width; (D) Number (#) of goblets per villi and area of the villi. (E) Representative images of haematoxylin-eosin stains of the intestine. CTR: Control group; HYP: acute hypobaric hypoxia group. *n* = 9–10 per group. * *p* < 0.05 vs. CTRL. Statistical analysis was performed using Student’s t-test to compare CTR and HYP groups. Differences were considered statistically significant when *p* < 0.05. The box represents the interquartile range, showing the first and third quartiles separated by the median; the mean value is indicated inside the box by a cross. Whisker endpoints represent the minimum and maximum values

To evaluate the changes in the small intestine histology, different parts of the intestinal mucosa were analysed. Regarding the intestinal crypt analyses, a significant reduction in the crypt depth was found in the HYP group (193 ± 7 μm vs. 163 ± 12 μm, *p* = 0.031) (Fig. 2A). Moreover, no statistically significant differences were found in the number of goblet cells counted in the crypts (5.81 ± 0.79 vs. 8.50 ± 1.30, *p* = 0.145), but a non-significant reduction in the size of these goblets was observed (62.0 ± 4.8 µm^2^ vs. 51.4 ± 3.8 µm^2^, *p* = 0.074) (Fig. 2B). On the other hand, in the intestinal villi analysis, no changes between CTR and HYP groups were found in any of the morphological parameters of the villi measured (villi height: 371 ± 76 µm^2^ vs. 347 ± 44 µm^2^, *p* = 0.407 and villi width: 118 ± 23 µm^2^ vs. 111 ± 17 µm^2^, *p* = 0.536 (Fig. 2C); villi area: 41.9 ± 13.2 µm^2^ vs. 34.2 ± 9.7 µm^2^, *p* = 0.167 (data not shown)) nor in the number and size of the goblets cells founds in the villi (*p* = 0.884 and *p* = 0.606, respectively) (Fig. 2D).

#### Caecal bacterial subgroups

**Fig. 3:**
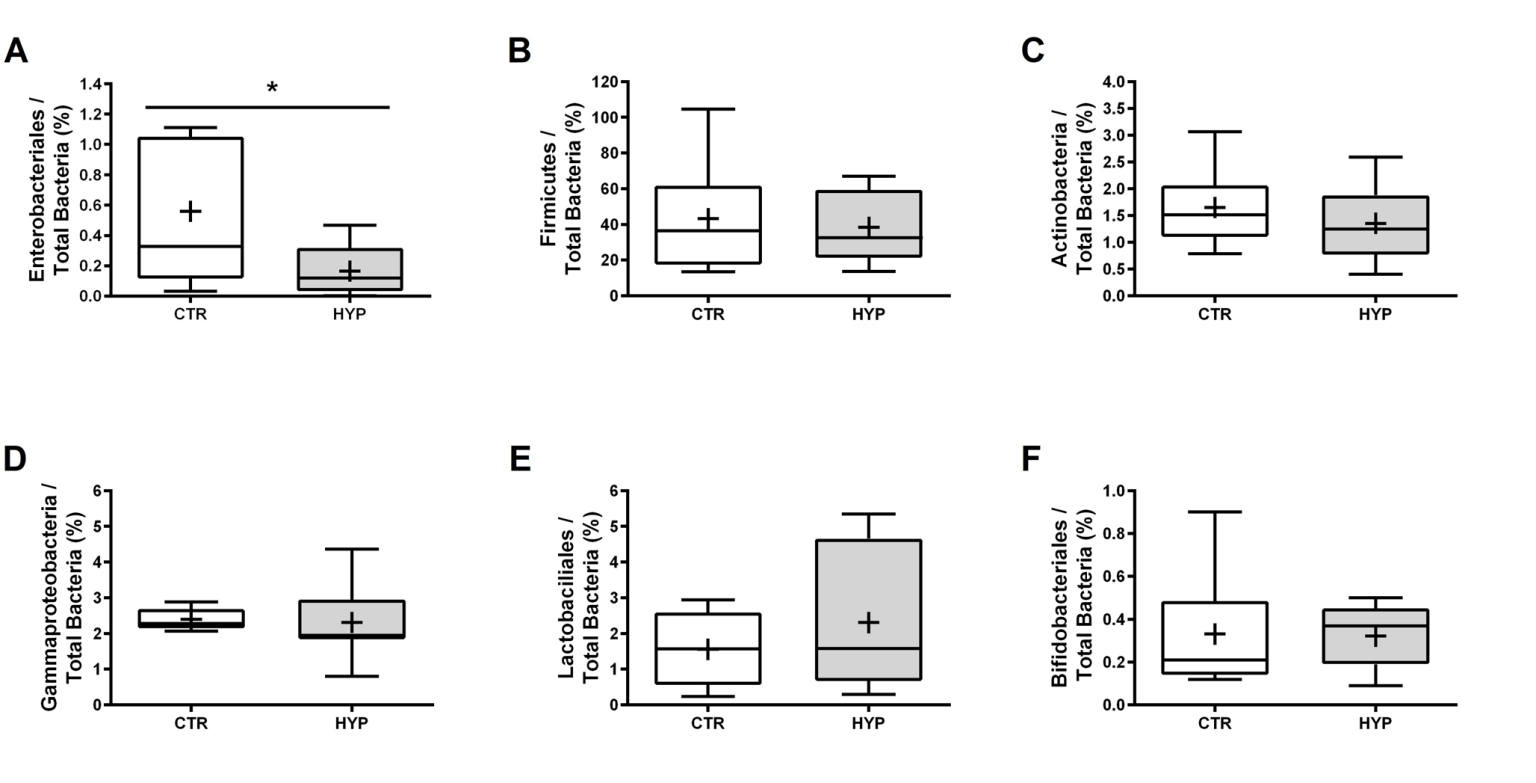
Effects of acute exposure to hypobaric hypoxia on caecal gut bacteria. (**A**) Enterobacteriales; (**B**) Firmicutes; (**C**) Actinobacteria; (**D**) Gammaproteobacteria; (**E**) Lactobacilliales; (**F**) Bifidobacteriales. CTR: Control group; HYP: acute hypobaric hypoxia group. *n* = 9–10 per group. * *p* < 0.05 vs. CTRL. Statistical analysis was performed using Student’s t-test to compare CTR and HYP groups. Differences were considered statistically significant when *p* < 0.05. The box represents the interquartile range, showing the first and third quartiles separated by the median; the mean value is indicated inside the box by a cross. Whisker endpoints represent the minimum and maximum values

The relative populations of the different bacterial subgroups were evaluated in caecal samples after acute hypobaric hypoxia exposure (Fig. 3). Total bacteria proportion in the caecum was similar between the studied groups (CTR = 35.8 ± 10 vs. HYP = 48.8 ± 26.8, *p* = 0.170). However, a statistically significant reduction on Enterobacteriales was shown after 4 h of hypobaric hypoxia exposure (*p* = 0.030) (Fig. 3A). At the phylum level, no changes were observed in Firmicutes or Actinobacteria (*p* = 0.780 and *p* = 0.380, respectively) after the hypoxic stimuli (Fig. 3B and C). In this same line, no changes were observed at the class level, in Gammaproteobacteria (Fig. 3D). In the order of the bacteria Lactobacilliales and Bifidobacteriales were unchanged in HYPO hypoxia (*p* = 0.314 and *p* = 0.478, respectively) (3E and F). Finally, no differences were found in *Prevotella* proportion (CTR = 0.567 ± 0.350 vs. HYP = 0.614 ± 0.329, *p* = 0.767) (data not shown).

#### Caecal short chain fatty acids

The analysis of the caecal SCFAs (Table 3) revealed that acute hypobaric hypoxia produced non-significant reductions in two of the most abundant SCFAs, acetic acid and propionic acid. However, no changes were observed in butyric acid, the other of the major SCFAs. Similarly, no changes were found in isobutyric, isovaleric, and valeric acids.

**Table 3:** Effects of acute exposure to hypobaric hypoxia on Short- Chain Fatty Acid (SCFA) caecal content of rats

Compound	CTR		HYP		
	Mean	SD	Mean	SD	*P* value
Acetic acid	85.5	7.13	63.5	8.0	*p* = 0.084
Propionic acid	27.1	1.52	20.9	3.8	*p* = 0.062
Isobutyric acid	3.22	0.13	3.29	0.43	*p* = 0.660
Butyric acid	15.5	1.83	15.0	3.0	*p* = 0.934
Isovaleric acid	3.41	0.24	3.73	0.52	*p* = 0.487
Valeric acid	3.37	0.31	3.05	0.54	*p* = 0.447

Data are presented as mean (mmol/kg) ± standard deviation (SD). *CTR* control group, *HYP* acute hypobaric hypoxia group. *n* = 9–10 per group. Statistical analysis was performed using Student’s t-test to compare CTR and HYP groups. Differences were considered statistically significant when *p* < 0.05

### High-resolution respirometry: Skeletal muscle mitochondrial respiration and reactive oxygen species production

**Fig. 4:**
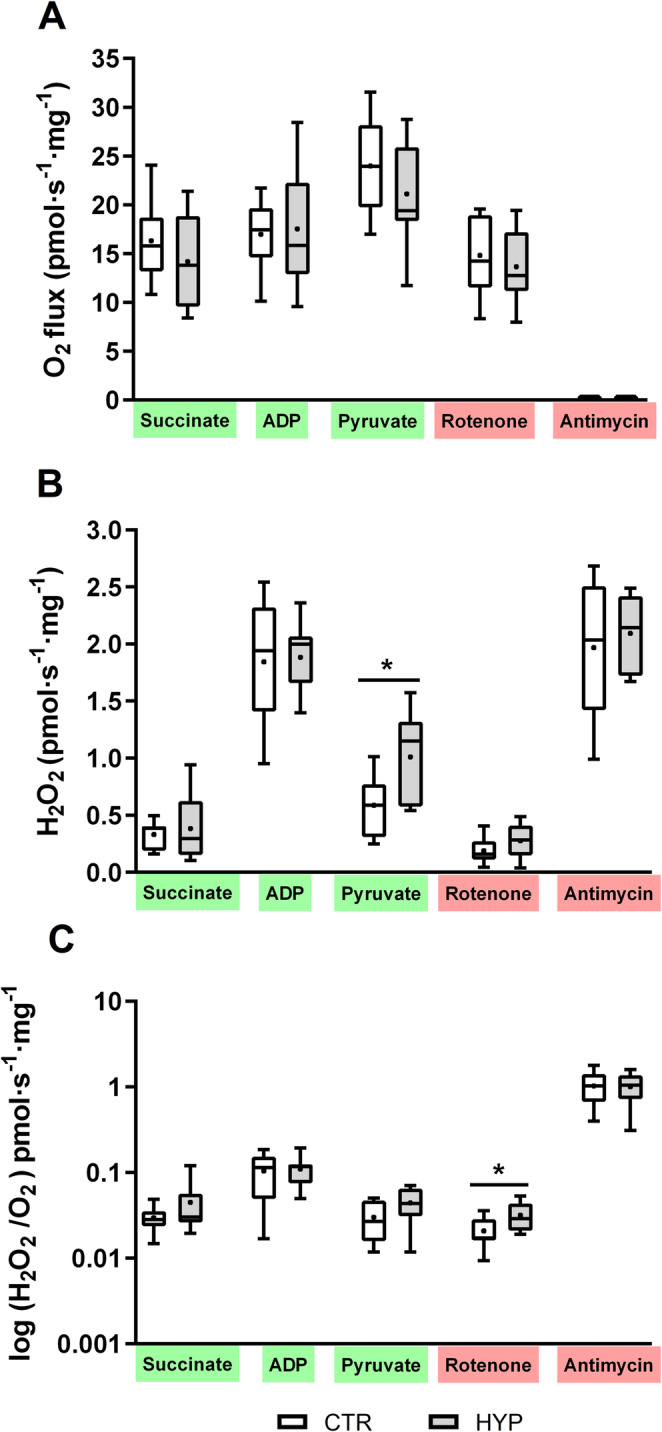
Effects of acute exposure to hypobaric hypoxia on soleus muscle mitochondrial respiration and oxygen reactive oxygen production. (**A**) O_2_ consumption; (**B**) H_2_O_2_ production (**C**) H_2_O_2_/O_2_ ratio. The substrates (Succinate, ADP and pyruvate) are identified with the green colour and inhibitors (rotenone and antimycin) with the red colour. CTR: Control group; HYP: acute hypobaric hypoxia group. *n* = 9–10 per group.* *p* < 0.05 vs. CTRL. Statistical analysis was performed using Student’s t-test to compare CTR and HYP groups. Differences were considered statistically significant when *p* < 0.05. The box represents the interquartile range, showing the first and third quartiles separated by the median; the mean value is indicated inside the box by a dot. Whisker endpoints represent the minimum and maximum values

Mitochondrial respiration and H_2_O_2_ production in muscle fibres were measured by high-resolution respirometry. No differences between groups were found in O_2_ consumption in any of the analysed respiratory states (Fig. 4A). However, regarding H_2_O_2_ production, a significant increase was found in the HYP group after adding pyruvate (complex I) (*p* = 0.033) (Fig. 4B). Finally, the ratio between H_2_O_2_ production and O_2_ consumption (Fig. 4C) indicates a statistically higher H_2_O_2_/O_2_ ratio in the HYP group after the addition of rotenone and consequent inhibition of mitochondrial complex I.

## Discussion

### Biometric and haematological responses to acute hypobaric hypoxia

Exposure to hypobaric hypoxia is characterised by “altitude anorexia”, resulting from a combination of factors including body weight loss (muscle mass and fat mass reduction), increased metabolic rate, decreased food intake, and water loss suffered during hypoxic exposure [32–34]. However, the extent of body weight and muscle mass reduction is directly influenced by the type of exposure (acute, chronic, or intermittent), the duration of the stay, the altitude level, the intensity of the physical exercise performed (energy expenditure), and the individual adaptive responses to hypoxia [33]. It has been reported that prolonged exposures to high altitudes tend to reduce body weight in both animals and humans, whereas intermittent exposures over shorter periods preserve body weight and muscle mass [32, 35–37]. These previous observations align with our findings, which demonstrate that, despite the acute exposure to severe hypoxia (5,000 m), a short-term duration (4 h), such as the one applied in the present study, preserves most tissues and organs from a serious catabolic distress [38] (Table 2).

The reduction in tissue oxygen levels activates hypoxia inducible factor-1α (HIF-1α) leading to increased erythropoietin (EPO) production, thereby enhancing the oxygen-carrying capacity of the blood. It has been previously reported that 90 min of hypoxic exposure (5,000 m) is sufficient to induce significant increases of EPO, which is detectable in blood 180 min post-exposure [39]. EPO is primarily produced in the kidneys and, through its receptor (EPO-R) in bone marrow, stimulates the proliferation and differentiation of the specific erythroid progenitor cells, which is finally translated into the production of new mature RBCs [39–41]. In our investigation, blood samples were collected from the animals immediately after the hypoxic stimulus. Therefore, while it is plausible that the hypoxic exposure initiated the signalling pathways leading to increased EPO secretion, the short plasma half-life of signalling molecules such as EPO or VEGF, and the timing of the sampling precluded the detection of increases in circulating RBC, haemoglobin, and haematocrit (Fig. 1).

Acute exposure to high altitude is associated with an increase in stress hormones such as cortisol, adrenaline, and noradrenaline. However, acclimatisation to altitude through continuous stay in a hypoxic environment leads to the normalisation of plasma cortisol and adrenaline levels [42]. Considering that stress hormones also influence WBC counts, the leukocyte response to altitude includes a rapid increase in lymphocytes within the first 30 min, followed by a loss of neutrophils and lymphocytes (neutrophilia and lymphopenia) observed between 2 and 4 h of exposure [42]. HIF-1 plays a crucial role in maintaining innate and adaptive immune homeostasis during physiological and acute hypoxia, contributing to the differentiation and activation of leukocytes, and allowing them to translocate to target tissues and perform their functions [43]. Our results demonstrated a 24% reduction in the number of leukocytes after 4 h of AHH, accompanied by a non-significant 16% increase in lymphocytes and a 14% decrease in granulocytes (Fig. 1). In this line, a previous study, in which prolonged hypobaric hypoxia exposure (28 days at 5,000 m) was used, also reported a reduction in leukocytes [44]. Nonetheless, proper acclimatisation to altitude results in the return of WBC counts and stress hormones to baseline [42]. While, in contrast, chronic pathophysiological hypoxic conditions can lead to immunosuppression and immune system imbalance [43].

Several studies have reported a significant reduction in platelet count following various forms of hypoxic exposure, including acute hypobaric hypoxia [45], intermittent hypobaric hypoxia (9 days at 4,500 m) [35] and chronic hypoxia [46]. This reduction in circulating platelet count is often attributed to increased platelet activation [45, 47]. It is considered that the reduced platelets count in hypoxia is related to the higher activation and consumption of platelets due to increased polycythaemia, and due to the increase in platelet capture and destruction in pulmonary vessels - when there is a pathophysiological state of lungs, as pulmonary infection or pulmonary hypertension, a common occurrence in high altitude exposure [46]. In contrast to these findings, we did not observe changes in platelet count following our hypoxic exposure protocol (Fig. 1). This discrepancy may be due to the short duration of the hypoxic exposure (4 h), which might not have been sufficient to induce erythrocytosis or pulmonary hypertension, both of which are associated with increased platelet reduction. Furthermore, the timing of blood sampling, immediately after hypoxic exposure, may have prevented the onset of significant haematological changes, such as alteration in RBC count, which are known to influence platelet dynamics.

### Intestinal and gut microbiota responses to acute hypobaric hypoxia exposure

#### Intestinal morphology

Like other tissues, the intestinal barrier can be compromised by rapid ascent to high altitude [43]. The intestine is vulnerable to the effects of hypoxia, due to its specialised blood supply and anatomical structure [48]. A damaged intestinal barrier leads to impaired nutrient absorption, which could in part explain the weight loss observed after chronic hypoxia [18]. Moreover, the GI damage could contribute to decreased physical and cognitive performance during acute exposure to hypoxia, and further, could play a role in the development of diseases associated with chronic intermittent hypoxia [16].

Previous studies have demonstrated that rats exposed to simulated altitudes of 4,000 and 7,000 m for 72 h exhibited bacterial translocation to organs such as the liver, spleen and lymph nodes, accompanied by increased serum endotoxin levels [48, 49]. Moreover, at the microscopical level, animals maintained above 4,000 m for 3 days displayed thinner intestinal epithelial mucus layer, reduced villi area (height and width), decreased surface area, irregular villi arrangement, inflammatory cell infiltration, and red blood cell exudation [48, 49]. In contrast to these findings, in this study we did not observe changes in villi morphology (length, width, and area), nor in the number of goblets per villi, nor in their size (Fig. 2), which may be related to the difference in the exposure time (4 h). These results suggest that functions like luminal sensing, digestion, absorption, secretion, and immune defence may remain uncompromised after acute hypoxic exposure [50]. However, Ma et al. (2023) reported that rats exposed to 7,000 m for 72 h exhibited intestinal epithelium deformation, reduced crypt depth, and a decrease in the abundance of goblet cells, suggesting a disruption of the mucus layer and exacerbated intestinal damage. Our results align with those of Ma et al. (2023), showing a significant reduction in crypt depth in rats submitted to AHH (Fig. 2). The microscopic structure of the intestine, particularly crypt depth, serves as an indicator of intestinal development and health [51]. The intestinal crypt is the site where various intestinal cells, such as epithelial cells, goblet and mast cells, are present. These cells play crucial roles in maintaining epithelial integrity, ensuring intestinal homeostasis, and supporting immune response [3, 51]. It appears that a 4 h exposure to 5,000 m is sufficient to induce certain modifications in gastrointestinal structure, such as reduction of crypt depth. However, it remains unclear whether these responses are severe enough to cause significant intestinal injury, such as a long-term reduction in goblet cell formation, as observed with more prolonged exposures (e.g. 3 days at 7,000 m) [3]. Although we observed a trend toward smaller goblet cell area, the total number of goblet cells within crypts remained unchanged (Fig. 2). Goblet cells secrete mucins to form the mucosal layer, preventing direct contact between luminal content and epithelial cells, thereby contributing to the preservation of the intestinal barrier [18]. A reduction in goblet cell area could directly impair mucin secretion, potentially contributing to GI damage, while a reduction in their number leads to diminished intestinal mucus secretion, and therefore, to a thinner or discontinuous intestinal mucus layer. These mucus lining alterations increase the permeability of the intestinal barrier, facilitating the translocation of endotoxins and pathogens [3, 51].

### Microbial communities

As it has been discussed in previous paragraphs, environmental hypoxic exposure can induce different pathological changes in the intestinal tract, such as intestinal mucosal damage, atrophy, and barrier dysfunction. These alterations can directly impact intestinal microecology and, consequently, host health [52]. Aerobic and facultative anaerobic bacteria are the most dominant in the oxygen-rich environment of the stomach. In contrast, the small-intestine and large intestine, which experience progressively lower oxygen levels, are primarily colonised by facultative and obligate anaerobes, respectively [2]. Thus, the colon is dominated by a community of obligate anaerobic bacteria (dominated by phyla Firmicutes and Bacteroidetes). These bacteria are beneficial because they possess a vast array of enzymes for hydrolysing different complex carbohydrates. However, the colon also harbours facultative anaerobic bacteria (phylum Proteobacteria), which are not specialised in fibre degradation and could even metabolise fermentation products into carbon dioxide when oxygen is present [21]. Previous research indicates that days 5 and 7 may be critical time points to observe substantial impacts on gut microbiota in animals exposed to altitudes ranging from 2,800 m and 5,500 m [52, 53]. Certainly, after 7 days of exposure to either moderate or high altitudes, a reduction in the proportion of aerobic bacteria and an increase in anaerobic bacteria within the gut microbiota have been reported [52–54]. In our study, significant change was observed in Enterobacteriales, a facultative anaerobic bacterium order, which showed reduced abundance in response to decreased oxygen pressure (Fig. 3A).

Previous studies have reported that 7 days of moderate (2,800 m) or high-altitude (4,300 m) exposure led to an increased abundance of Firmicutes in rats [52]. A similar trend, characterized by an increase in Firmicutes and a decrease in Bacteroidetes, has also been observed in both high-altitude native populations and lowlanders acclimated to altitude (3,100 m) [55]. An elevated Firmicutes/Bacteroidetes ratio is commonly associated with hypoxic exposure [3, 19, 44], and is often accompanied by a higher Bacteroides/*Prevotella* ratio [44]. However, findings across studies are inconsistent because other researchers have reported a reduction in the abundance of Firmicutes and an increase in Bacteroidetes following 24 h [44, 46] and 5 days (5,500 m) [53] of altitude exposure, with these changes becoming more pronounced after 5 weeks (Han et al., 2022). In the present study, after 4 h of HH (5,000 m), there were no changes in Firmicutes population, which reiterates the variability of response of this population to a situation of hypoxia.

Short-term exposure (5–7 days) to moderate altitude (2,800 m) has been shown to reduce the relative abundance of Proteobacteria. In contrast, more severe hypoxic conditions (4,300 m − 5,500 m) have been linked to an increased relative abundance of Actinobacteria [52]. The genus *Prevotella* exhibits a time-dependent response to hypoxia. Studies have shown a decrease in *Prevotella* abundance after hypoxic stimuli that lasts between 24 h and 28 days [44, 46]. However, prolonged exposures (5 weeks) [53] and observations in wild animals and human populations native to high altitudes tend to exhibit a higher abundance of *Prevotella* [44, 46, 53]. Moreover, it is important to highlight that *Prevotella* has been described as a key bacterial genus involved in the symptoms of Acute Mountain Sickness and hypoxia acclimatisation [46, 56]. However, in our study, we did not observe changes neither in Proteobacteria (Gammaproteobacteria), Actinobacteria nor in *Prevotella*.

Despite some studies have detected changes as early as 24 h after exposure to 5,500 m [57], in our study the only significant change in gut bacterial populations was observed in Enterobacteriales. This suggests that longer exposure durations may be necessary to elicit detectable alterations at the level of microbial family abundance [52]. The absence of significant changes in our results does not imply a lack of microbial community response. It is possible that the changes were too subtle to be detected at our observation time point, or that hypoxic signalling requires more time to induce measurable shifts in the bacterial community.

#### Short chain fatty acids

The large number of bacteria that reside in the GI tract, in addition to aiding in the digestion of the aliments, produce several vitamins and synthesise SCFAs [58]. SCFAs are the main metabolites found in the gut, resulting from the fermentation of nondigestible dietary fibres and resistant starch [59, 60]. Besides serving as the preferred metabolic substrate for epithelial cells, SCFAs are also transported through the bloodstream to other parts of the body [44, 58, 59]. Acetic acid, propionic acid, and butyric acid account for approximately 95% of the total SCFAs produced in the gut (Table 3) [2, 16, 59].

SCFAs are considered important mediators in the communication between the gut microbiota and skeletal muscle. The presence of the SCFA receptors GPR41 (G protein-coupled receptor 41) and GPR43 in skeletal muscle tissue supports the role of these volatile fatty acids in the gut–muscle axis [27, 61, 62]. SCFAs have been shown to increase AMPK (AMP-activated protein kinase) phosphorylation in myotubes and skeletal muscle, possibly by increasing intracellular AMP concentrations and, consequently, the AMP/ATP ratio [27, 61]. In this context, SCFAs promote fatty acid uptake and oxidation, enhance glucose uptake and glycogen synthesis, partly through increased GLUT4 activity and/or expression, while reducing lipogenesis [61], thereby improving insulin sensitivity [27, 61, 62]. Therefore, SCFA-induced AMPK phosphorylation appears to be a key mechanism underlying metabolic adaptations in skeletal muscle [27, 61, 63].

In addition, SCFAs can stimulate the release of hormones from different organs into the systemic circulation. These endocrine signals influence skeletal muscle metabolism through peptide and protein hormones such as glucagon-like peptide-1 (GLP-1), insulin, and leptin, which are involved in appetite regulation, insulin sensitivity, and glucose metabolism [61, 62]. Moreover, SCFA-induced gut-derived PYY secretion may indirectly contribute to improved insulin-mediated glucose uptake in skeletal muscle and overall glucose homeostasis [62]. Finally, SCFAs may exert indirect effects on skeletal muscle through their actions on the vascular system. For example, acetate has been reported to decrease vascular resistance and induce vasodilation [61]. Despite we did not show an increase in acetic acid (Table 3), this mechanism could be particularly interesting under hypobaric hypoxic conditions, where enhanced tissue perfusion may help ensure adequate oxygen delivery to the cells.

Less is known about the effects of hypobaric hypoxia on SCFA production, although it is believed to play a potential role in host acclimatisation to the altitude through the regulation of SCFA metabolism by gut microbiota [44]. The modification of gut microbial composition to enhance fermentative capacity may induce higher SCFA production, supporting the host´s ability to maintain energy homeostasis and metabolic efficiency at altitude [44]. Karl et *al*. (2018) reported a significant reduction in propionate levels and a trend toward decreased total SCFA production in humans after 28 days at high altitude (4,300 m) [56]. Similarly, in rats, 28 days of exposure to 5,000 m resulted in reduced levels of total SCFAs, particularly propionate and butyrate, along with an increase in acetate [44]. In contrast, Huang et *al.* (2021) observed increased levels of SCFAs—including acetate, propionate, and butyrate—after 4 weeks of normobaric hypoxia (16.4% O₂, equivalent to 2,000 m). These findings suggest that acute exposure to hypobaric hypoxia may reduce butyrate and propionate production, while chronic exposure may allow a recovery of these SCFAs, likely associated with shifts in microbial community composition [44]. In the present study, a 15 h fasting protocol was uniformly applied to both groups to ensure experimental standardization and eliminate the immediate confounding effects of recent food intake. However, it is worth noting that such a fasting duration substantially depletes fermentable substrates in the rat cecum, which typically drives baseline SCFA levels to a physiological minimum. Acute hypobaric hypoxia (HYP) led to non-significant reductions in acetic and propionic acids, while butyric, isobutyric, isovaleric, and valeric acids remained unchanged (Table 3). This lack of statistical significance could potentially be attributed to a masking or floor effect induced by the prolonged fasting state. With SCFA production already diminished due to nutrient deprivation in both groups, the window to detect further acute decreases driven by hypoxic stress was inherently limited. Nonetheless, the selective downward trend in the two most abundant SCFAs (acetic and propionic acids) suggests that early hypoxia-mediated disruptions, such as altered mucosal perfusion or shifts in luminal pH, were beginning to unfold, even against the backdrop of a nutrient-depleted caecal microenvironment.

### Skeletal muscle mitochondrial respiration and reactive oxygen species production

Acute hypobaric hypoxia appears to increase electron leak under Complex I-supported respiration (manifested as higher H₂O₂ with pyruvate), consistent with enhanced ROS generation via reverse electron transfer under reduced quinone pools [64]. When Complex I was inhibited by rotenone, ROS production tended to increase while O₂ consumption was maintained, resulting in an elevated H₂O₂/O₂ ratio — possibly due to sustained ROS generation at downstream sites such as Complex III or other electron leak pathways [65, 66]. Interestingly, despite these alterations in ROS production, mitochondrial O₂ consumption was unchanged across all respiratory states, suggesting, in contrast with Magalhães et al. (2005), preserved oxidative phosphorylation capacity, while redox efficiency was reduced [67]. This suggests a shift toward higher electron leak without impairing the electron transport chain’s capacity to consume oxygen, supporting the view that acute hypobaric hypoxia induces qualitative, rather than quantitative, mitochondrial alterations.

Among SCFAs, butyrate appears to play a particularly important role in the activation of PGC-1α [27]. Given the central role of PGC-1α in mitochondrial biogenesis and function, its upregulation by SCFAs may promote a more oxidative skeletal muscle phenotype while enhancing mitochondrial fatty acid β-oxidation and OXPHOS capacity [27, 61]. Notably, as referred to before, no significant changes were observed neither in the gut microbiota composition nor in the SCFAs under acute hypoxia, suggesting that the early mitochondrial responses in skeletal muscle seem to occur independently of gut-derived signals. Taken together, findings appear to show that initial adaptations to hypobaric hypoxia are primarily intrinsic to the muscle, with potential contributions from the gut-muscle axis likely emerging only under more prolonged or severe hypoxic conditions.

In summary, in our study we did not observe changes in body weight, organ proportions, and haematological parameters, indicating that the exposure was too brief to trigger full adaptive responses such as erythropoiesis. The intestine appears to be the first organ structurally affected by hypoxia, as indicated by a significant reduction in crypt depth, although villus morphology and goblet cell counts were preserved. Intestinal microbiota seems to be also early affected, as demonstrated by a significant decrease in abundance of Enterobacteriales. Other bacterial groups and short-chain fatty acid (SCFA) levels remained stable. This indicated that more dramatic microbial shifts may require longer exposure durations. Finally, an increase in reactive oxygen species (ROS) production was detected in skeletal muscle while mitochondrial respiration was unaffected. This indicates a qualitative shift in redox efficiency without compromising oxygen consumption. These results suggest that initial adaptations to acute hypoxia are primarily intrinsic to muscle tissue, with potential gut–muscle axis interactions likely emerging under more prolonged or intense hypoxic conditions.

The main limitation of this study is that it was conducted in a laboratory rat model. It is obvious that, for ethical and technical reasons, it is virtually impossible to perform this study in humans. However, although sensitivity to hypoxia differs between the two species, they share the same basic mechanisms of action of hypoxia at the cellular level, therefore, the information obtained has intrinsic interest and value from a basic physiology approach. Another limitation stems from the differences in the composition of the gut microbiota between the two species, which does not allow for a direct extrapolation to human clinical practice.

## Conclusions

Acute exposure to severe hypobaric hypoxia (4 h at 5,000 m) in rats did not produce major physiological, haematological, intestinal, microbial, or muscular disruptions. While a reduction in intestinal crypt depth and increased mitochondrial ROS production were observed, most parameters—including gut microbiota composition and muscle mitochondrial respiration—remained stable. These findings suggest that short-term hypoxic stress may trigger early, localized responses without compromising systemic homeostasis, and that more prolonged or repeated exposures may be required to elicit broader gut–muscle axis adaptations. 

## Data Availability

No datasets were generated or analysed during the current study.
